# Rapid nitrogen loss from ectomycorrhizal pine germinants signaled by their fungal symbiont

**DOI:** 10.1007/s00572-020-00959-7

**Published:** 2020-05-03

**Authors:** Joshua M. Smith, Matthew D. Whiteside, Melanie D. Jones

**Affiliations:** 1 Biology Department and Okanagan Institute of Biodiversity Resilience and Ecosystem Services, University of British Columbia, Okanagan campus, Kelowna, British Columbia V1V 1V7 Canada; 2 Present Address: Xeriscape Endemic Nursery & Ecological Solutions, West Kelowna, British Columbia V1Z 1Z9 Canada

**Keywords:** Ectomycorrhizas, Nitrogen, Nitrogen priming, Nutrient transfer, Seedling establishment, *Pinus contorta* (lodgepole pine)

## Abstract

Ectomycorrhizal fungi supply their plant partners with nitrogen but can also retain substantial amounts. The concentration of nitrogen in the soil and the amount of carbon supplied from the host seem to influence the proportion of N retained by the fungus. In an experiment designed to determine whether differential supply of nitrogen to two plants influenced nitrogen transfer from fungus to plant within a mycorrhizal network, we observed rapid, substantial loss of nitrogen from pine seedlings. The loss occurred when the mycorrhizal fungus experienced a sudden increase in nitrogen supply. We grew *Pinus contorta* seedlings in association with *Suillus tomentosus* in low-nitrogen microcosms where some nitrogen was accessible only by hyphae. After 70 days, foliage of some seedlings was treated with nitrogen. Three days later, hyphal nutrient media were replaced with water or a solution containing nitrogen. Foliar treatment did not affect nitrogen transfer by the fungus to shoots, but by day 75, seedling nitrogen contents had dropped by 60% in microcosms where nitrogen had been added to the hyphal compartments. Those seedlings retained only 55% of the nitrogen originally present in the seed. Loss of nitrogen did not occur if water was added or the hyphae were severed. Because of the severing effect, we concluded that *S. tomentosus* triggered the loss of seedling nitrogen. Nitrogen may have been lost through increased root exudation or transfer to the fungus. Access to nitrogen from nutrient-rich germinants would benefit rhizosphere microorganisms, including ectomycorrhizal fungi colonizing pine from spores after wildfire.

**Electronic supplementary material:**

The online version of this article (10.1007/s00572-020-00959-7) contains supplementary material, which is available to authorized users.

## Introduction

In boreal and montane forests of the Northern Hemisphere, where nitrogen (N) is typically deficient (Hunt et al. 1988; Brockley 2001b; Högberg et al. 2017), ectomycorrhizal (EM) fungi mineralize organic forms of N and translocate a portion of the solubilized N to their plant partners (Bending and Read 1995; Perez-Moreno and Read 2000; Högberg et al. 2007; Nicolás et al. 2019). Although EM fungi can transfer substantial amounts of N to their hosts (Hobbie and Högberg 2012; Akroume et al. 2019), they retain varying amounts of N to meet their own needs (Hobbie et al.2008; Näsholm et al. 2013). Sometimes such a high proportion of N is reserved by the fungus that plant growth is negatively affected (Abuzinadah and Read 1989; Colpaert et al. 1996; Corrêa et al. 2008; Franklin et al. 2014). Several factors influence the proportion of absorbed N that EM fungi transfer to their plant symbionts. For example, increased fungal sink strength can result in higher EM fungal N and reduced transfer to the host (Bidartondo et al. 2001; Alberton et al. 2007; Hasselquist et al. 2016). By contrast, EM fungi tend to transfer a higher portion of absorbed N when N supply is higher (Corrêa et al. 2008; Alberton and Kuyper 2009; Näsholm et al. 2013). The form of N supplied and the EM fungal species present will also influence N transfer to the plant (Abuzinadah and Read 1989; Hobbie et al. 2008; Alberton and Kuyper 2009; Jones et al. 2009).

The question of which factors influence the proportion of absorbed N that is subsequently transferred from mycobiont to phytobiont is especially interesting when considering mycorrhizal networks. In ectomycorrhiza-dominated forests, many trees are part of mycorrhizal networks, where an individual plant associates with many fungal individuals and, in turn, each fungal genet may associate with multiple plants (Simard and Durall 2004; Beiler et al. 2010). Models generated by Franklin et al. (2014) suggest that EM fungi will invest more in N uptake and transfer when competing with other EM fungi on a root system and that plants will allocate more C to their fungal symbionts when the fungi are associated with other hosts. Although one might expect that a networked EM fungus would translocate more N to a host that supplies relatively more C, experiments on individual EM plants indicate either that C and N transfers between symbionts are not quantitatively related (Corrêa et al. 2008) or that increased belowground C allocation from the plant results in reduced N transfer to the tree (Hasselquist et al. 2016). Furthermore, there is no indication that EM plants “reward” fungi that supply more N by allocating more carbon to them (Hortal et al. 2017). Instead, as described above, N supply rate has a greater influence on the amount of N supplied to an EM plant by its fungal symbiont. The effect of differences in plant nutrient status within an EM network on nutrient transfer from the fungus to networked plants is not known, however.

In the study described here, we took advantage of the fact that pine seedlings can absorb nutrients through their foliage (Uscola et al. 2014) to supply different amounts of N to two pine seedlings associated with the same fungus. Because higher foliar N is generally correlated with higher carbon assimilation rates (Evans (1989)) and Hasselquist et al. (2016) found higher photosynthetic rates resulted in greater belowground carbon allocation and reduced N transfer to 15-year-old *Pinus sylvestris*, we hypothesized that a networked EM fungus would allocate more N to a plant partner with lower tissue N concentrations than one with higher tissue N. We assembled Petri plate microcosms containing two *Pinus contorta *var. *latifolia* Engelm. seedlings ectomycorrhizal with *Suillus tomentosus*, a fungus that is common on lodgepole pine roots in lower N sites (Kranabetter et al. 2006; Jones et al. 2012). Fungal hyphae, but not roots, could access N in a small well within the microcosms. Part way through the experiment, we labeled the wells with ^15^N to follow its transfer from EM fungus to seedling. While the ^15^N distribution results were inconclusive, we observed a very rapid and substantial loss of N by the seedlings in response to an increase in N supply to the fungus; that observation is the focus of this paper.

## Materials and methods

### Preparation of fungal material and seedlings

*Suillus tomentosus* (Kauffman) isolated from a local young lodgepole pine stand was re-cultured on solid 1/10 Modified Melin-Norkrans (MMN) media (Marx and Bryan 1975) and grown for 1 month. Then ~ 1 cm^3^ pieces of medium, containing a distal edge portion of the hyphae, were placed individually on 1 × 8 cm pieces of charcoal-infused filter paper (#728, Macherey-Nagel GmbH and Co. KG), plated on 1/10 MMN agar. Fungi were allowed to grow for 1 month, after which they were used in the microcosms. *Pinus contorta* Dougl. var. *latifolia* Engelm. seeds (Seedlot 30,556, British Columbia Ministry of Forests, Lands and Natural Resource Operations, Tree Seed Center, Surrey, British Columbia, Canada, FORHTIP.TreeSeedCentre@gov.bc.ca) were surface sterilized in aerated 30% hydrogen peroxide for 15 min and then rinsed in aerated sterile deionized water overnight. The seeds were then plated on sterile water agar (8%) and kept at a constant 22 °C, with a 12-h photoperiod of 370 μmol m^−2^ s^−1^ photosynthetically active radiation (GC-20, Biochambers Inc. Winnipeg, MB) for 1 month.

### Assembly of microcosms

**Fig. 1:**
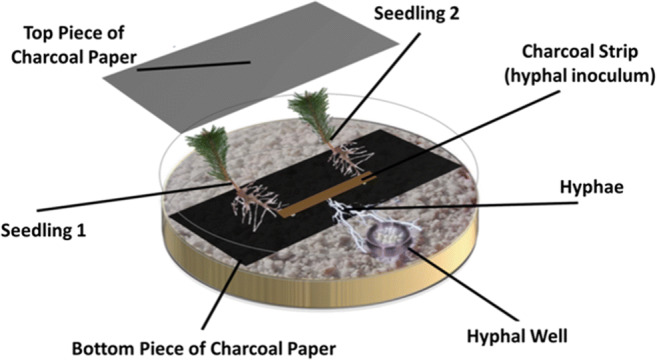
Diagram of microcosms containing two *Pinus contorta* seedlings growing in association with a single *Suillus tomentosus* colony on a low-N agar medium overlain with a layer of perlite. The hyphal well initially contained high-N media overlain with perlite and was accessible by only hyphae

Each microcosm contained a well, which was accessible only to the hyphae and contained higher concentrations of N than the rest of the media. To prepare these wells (Fig. 1), caps were removed from autoclaved 1.5-ml microcentrifuge tubes and secured to the bases of empty Petri dishes (10-cm diameter), 2 cm away from the plate edge, by gently melting the cap with a soldering iron. Each plate was then filled with 20 ml of pH 5.7, ¼ strength solid MMN media containing no glucose or malt and with 1/40 the concentration of N (0.095 mM NH_4_Cl) typically used in full-strength MMN. Each well was filled with 300 μl of pH 4.5, ¼ strength MMN media containing no glucose or malt, with 2.5 X the concentration of N (9.46 mM NH_4_Cl) of full MMN, and solidified with Phytagel™ (15 g l^−1^; Sigma Chemical Co. St. Louis). The relative concentrations of N in the main plate compartment versus the well were chosen to encourage hyphal proliferation in the well and to make seedlings more dependent on the EM fungus for access to N. The media in the plate were below the rim of the hyphal well, creating an air gap between the plate media and the well media, which prevented diffusion from the well to the rest of the media.

Autoclaved 6 × 8 cm rectangles of charcoal-infused filter paper were placed on the plate media, and then sterile sieved Perlite™ (0.25–0.5 cm, The Scotts Company, Mississauga, Canada) was spread, one granule thick, over the media that remained exposed in the plate to provide an aerated growth surface for *S. tomentosus*, as per Crahay et al. (2013). A *S. tomentosus* inoculum strip (described in *Preparation of fungal material and seedlings*) was placed face-up in the middle of the filter paper rectangle (Fig. 1). Then two 1-month-old *P. contorta* seedlings were placed on each plate so that their roots were in contact with the inoculum (Fig. 1). A second 6 × 8 cm rectangle of autoclaved filter paper was placed on top of the *P. contorta* roots and inoculum strip so that it overlapped with the bottom rectangle piece. Sterile perlite was carefully added to the hyphal well, one granule thick, while ensuring that no contact was formed between the well media and the plate media. The plates were then sealed with Parafilm™ and the root section wrapped in aluminum foil. The microcosms were placed in the growth cabinet at a 45° angle under the same light and temperature conditions described in *Preparation of fungal material and seedlings*.

### Foliage and well treatments

At intervals, plates were removed from the growth cabinet and visually inspected to look for the presence of hyphae on the roots and in the hyphal wells. Both were present at approximately 40 days. Once the fungus had colonized the pine roots and wells for about a month, foliage and well treatments were initiated. Two treatments were applied to the foliage on day 70: (i) one shoot received soluble N and the other received H_2_O (Table 1) or (ii) both shoots received H_2_O. Three days later, in a full factorial design (Table 1), these foliar treatments were crossed with three well treatments: replacement of the original well media with (i) glycine, (ii) ammonium, or (iii) H_2_O. To quantify seedling N content and ^15^N natural abundance before adding fresh N to the wells, some microcosms were harvested on day 73, just before the hyphal well treatments were administered (Table 1). Some microcosms received both foliar and N well treatments but had any hyphae crossing into the well manually severed just minutes before N addition to the wells (referred to as the severing controls).

**Table 1:** Summary of experimental treatments

Treatment number	Foliar treatment	Hyphal well^c^	Harvest time (day)	Hyphae	Number of microcosms
1	Differential^a^	n/a	73	Intact	3
2	H_2_O^b^	n/a	73	Intact	3
3	Differential^a^	NH_4_	75	Intact	8
4	Differential^a^	Glycine	75	Intact	8
5	Differential^a^	H_2_O	75	Intact	4
6	H_2_O^b^	NH_4_	75	Intact	4
7	H_2_O^b^	Glycine	75	Intact	4
8	H_2_O^b^	H_2_O	75	Intact	4
9	Differential^a^	NH_4_	75	Severed	4
10	Differential^a^	Glycine	75	Severed	4

^a^Microcosms where the foliage of one of the two *P. contorta* seedlings was treated with 4.7 mM N-NH_4_Cl and the other received deionized H_2_O

^b^Microcosms where the foliage of both seedlings received H_2_O

^c^At day 73, 9.46 mM N-NH_4_Cl (98^+^ at% ^15^N), 9.46 mM N-glycine (99^+^ at% ^15^N), or deionized H_2_O were added to wells accessible by hyphae but not roots; n/a indicates treatments that were harvested before N additions were made to the hyphal wells

For foliage treatments, autoclaved 1.5-ml microcentrifuge tubes were filled with 800 μl of either sterilized deionized water or 4.7 mM N (NH_4_Cl) depending on the treatment (Table 1). The foliage of each plant was gently placed inside a microcentrifuge tube until it was approximately 75% submerged in the solution. Three days later, the microcosms were removed from the growth cabinet, and six microcosms were harvested (treatments 1 and 2 in Table 1; see below for procedure). The Phytagel™ media in the hyphal wells of the remaining microcosms was carefully dissolved with 50 mM citric acid and removed by pipette under a dissecting microscope, with as little disruption to hyphae as possible. Then isotopically labeled N was added to the wells to compare N allocation from the fungus to its two plant partners. Specifically, 400 μl of 9.46 mM 99^+^ at% ^15^N-glycine (Isotech Inc. Miamisburg, OH), 9.46 mM, 98^+^ at% ^15^NH_4_Cl (Cambridge Isotope Laboratories Inc. Andover, MA) or deionized water was added to each well (Table 1). The hyphae in four microcosms of the glycine and ammonium well treatments were manually severed with hot forceps where they entered the well. All microcosms were then placed back in the growth cabinet.

### Harvest

Forty-eight hours after initiation of the well treatments, the microcosms were harvested. The foliage treatment tubes were removed; then the tubes, intact seedlings, and microcosms were frozen separately at − 80 °C. Prior to freezing, all seedlings were rinsed with deionized water to remove surface N, especially residual N solution from the foliage treatment tubes. Once frozen, shoots were then separated from the roots. Plant tissues were dried at 75 °C for 5 days, weighed, and ground to pass through a 20-mesh screen using a Mini Wiley Mill (Thomas Scientific, Swedesboro, NJ) before determination of ^15^N–^14^N isotope ratio and total N via isotope ratio mass spectrometry (PDZ Europa ANCA-GSL elemental analyzer interfaced to a PDZ Europa 20-20 isotope ratio mass spectrometer [IRMS], Sercon Ltd. Cheshire, UK) at the University of California Davis, Stable Isotope Facility. In some instances, the root mass of individual seedlings was too low for mass spectrometry, so some replicates were combined (Online Resource 1).

### Nitrogen content of microcosm components and field seedlings

Nitrogen contents of all components of the microcosms were determined so that an N budget could be calculated. This included (a) eight samples of new charcoal-infused filter paper, (b) four samples each of the Perlite™ and agar media, and (c) six samples of *P. contorta* seeds from the seedlot used in the experiment. Sample preparation was identical to that described for experimental seedlings. Nitrogen was quantified with a Flash 2000 Elemental Analyzer (Thermo Fisher Scientific Inc.) at the British Columbia Ministry of Environment Technical Services Laboratory, Victoria, Canada (http://www2.gov.bc.ca/gov/content/environment/research-monitoring-reporting/research/analytical-lab). The amount of ammonium remaining in the microcentrifuge tubes applied to foliage was determined using the salicylate/nitroprusside colorimetric method of Kempers and Zweers (1986).

Additionally, to compare the tissue N concentrations observed in this study with those of first season, naturally establishing lodgepole pine seedlings, we sampled eight 16- to 20-week-old *P. contorta* seedlings from two recently disturbed clear-cut sites (50.09934°N 119.22346°W and 50.10418°N 119.22709°W, Beaver Lake Rd. near Kelowna, Canada). Seedlings were harvested whole and only gently manipulated to remove excess loose soil. The seedlings were then immediately transported to the lab where they were carefully rinsed to remove all soil and flash frozen at − 80 °C. Elapsed time between first seedling harvest and sample freezing did not exceed 2.5 h. Sample preparation of root and shoot tissues was identical to that described for experimental seedlings.

### Data analysis

One-factor ANOVAs, with %N in seedling tissues as the response variable, were conducted on shoots and roots from treatments (a) 1 and 2, to test for effects of neighbor treatments on shoot and root N before application of well treatments; (b) 6, 7, and 8, to test for well effects in the absence of differential foliar N treatments; and (c) 1 + 2 versus 9 + 10, to test for differences between seedlings harvested at day 73 with seedlings from the two severing control treatments. A test with foliage treatment as a fixed factor and plate as a random factor indicated no differences between foliage treatments for treatment 1 (*P* > 0.48; Online Resource 2); hence, the %N for the two seedlings per plate was averaged prior to these three analyses. Linear mixed models with hyphal well treatment and foliar treatment as fixed factors and plate as a random factor were used to compare (a) biomass of seedlings from the main harvest treatments (treatments 3–10); (b) %N of differential foliage treatments 3, 4, and 5; (c) %N of severed and unsevered N and water well treatments (treatments 3 + 4 vs 5 vs 9 + 10); and (d) at%^15^N of treatments 3, 4, and 5. When significant treatment effects were detected (*α* = 0.05), differences among means were determined using Tukey’s honest significant difference tests. Details of all linear models are presented in Online Resource 2. Because some seedlings were too small to generate sufficient tissue for analysis, tissues from two seedlings were combined for IRMS in some cases, reducing the number of replicates from those shown in Table 1 (Online Resource 1). A one-tailed *t* test was used to test whether there was net absorbance of N from the NH_4_ solutions applied to the foliage or whether there was net release of N by foliage into the tubes of water, in treatments 3, 4, 5, 6, 7, and 8.

Because residuals of at% ^15^N (treatments 3–8) were not distributed normally, yet there was no indication of effects of foliar treatments (*P* > 0.7) nor interactions between foliar and well treatments (*p* > 0.9), effects of well treatments were investigated using separate Kruskal-Wallis tests on at% ^15^N averaged across seedlings per plate for treatments 3–8, followed by Dunn multiple comparison tests. Kruskal-Wallis tests were also used to investigate differences in N content per seedling and effects of foliage treatment on at% ^15^N in treatments 3 and 4. All analysis were run in R version 3.6.1 (R Core Team 2019) except for the one-tailed *t* tests, which were run with JMP (V10, SAS Institute Inc. Cary, USA). Linear models were developed using *lm* and linear mixed models using *lmer* (package lmerTest).

## Results

### Distribution of ^15^N

At the main harvest, seedlings from treatments with intact hyphae and N additions to the wells (treatments 3, 4, 6, and 7, Table 1) were enriched in ^15^N compared to seedlings receiving water in the wells (treatments 5, 8; Online Resource 1, 3; Kruskal-Wallis for well treatment; shoots, *p* = 0.006; roots, *p* = 0.004). Labeling was evident in most, but not all seedlings: 65% of shoots and 93% of colonized roots from the labeled treatments were in excess of the upper 95% CI for unlabeled treatments. The foliar treatments had no effect on ^15^N enrichment (Online Resource 1, 3; Kruskal-Wallis for differential foliage treatment in treatments 3 and 4: shoots *p* = 0.4; roots *p* = 1.0). Although the results provide no evidence that N status of the seedlings influenced N translocation from the shared fungus, ^15^N enrichment of most shoots demonstrates that functional ectomycorrhizas had been established in which N absorbed from the wells was passed by hyphae to their plant partners.

### Nitrogen concentrations in seedling tissues

**Fig. 2:**
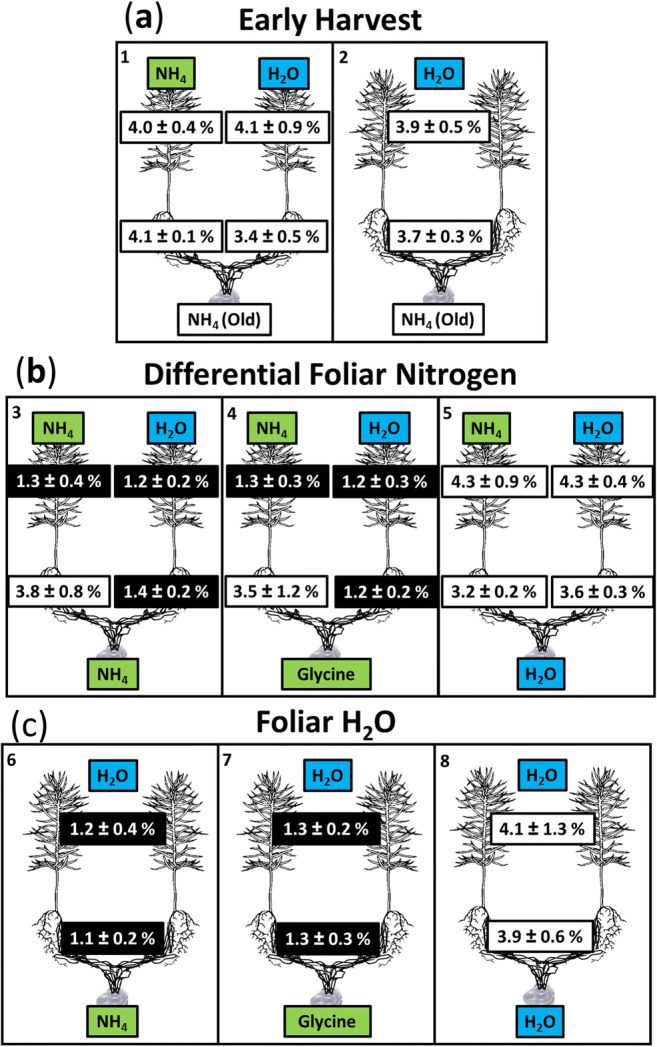
Nitrogen concentrations (%N; mean ± SD) of *P. contorta* roots and shoots in symbiosis with *S. tomentosus* after **a** 70-day growth followed by 3-day foliage treatment (H_2_O or 4.7 mM NH_4_, [*n* = 2–3]) or **b, c** an additional 2 days of exposure of *S. tomentosus* to fresh media in the hyphal well (H_2_O or 9.46 mM N from glycine or NH_4_Cl). Microcosms harvested at day 75 had either **b** different foliar N treatments to each seedling [*n* = 3–8] or **c** H_2_O applied to both seedlings [*n* = 6–8]. Within shoots and roots, figures shown in black are significantly different from those shown in white within treatment groups (ANOVA followed by Tukey’s HSD; Online Resource 2). Treatment numbers are in the top left corner of each box

Surprisingly, while the ^15^N results confirmed that some N had passed from fungus to plant over the 2 days after N was replenished in the wells, seedling N concentrations dropped dramatically over the same period in some treatments. On day 73, when seedlings had received the foliar N or H_2_O treatments for 72 h but the hyphal wells had not yet been refreshed, all seedlings had very high concentrations of N in both shoots and roots: close to 4% (Fig. 2a). By day 75, 48 h after solutions in the hyphal wells were refreshed, N concentrations in shoots had dropped by approximately 70% in microcosms where N had been added to the well (range 1.2–1.3 %N depending on treatment; treatments 3, 4, 6, 7; Fig. 2b, c). This occurred regardless of whether N was added as ammonium or glycine (Fig. 2b, treatments 3–5: well effect, *p* < 0.0001; foliar effect, *p* = 0.6; well x foliar, *p* = 0.9; Fig. 2c, treatments 6–8: well effect, *p* < 0.0001; Online Resource 2). Shoots of seedlings in microcosms that received water in the wells, rather than N, had no detectable change in N concentration (treatments 5, 8; Fig. 2b, c). Furthermore, pretreatment of the foliage with N did not affect the reduction in N concentration in the shoots (Fig. 2b), even though some N was absorbed by the foliage from the N-containing microcentrifuge tubes in some treatments (Online Resource 4). As with shoots, N concentrations in roots decreased rapidly in response to N addition to wells (Fig. 2c, treatments 6–8: well effect, *p* < 0.0001; Online Resource 2), but only in seedlings that did not have N applied to their foliage (Fig. 2b, treatments 3–5: well effect, *p* = 0.015; foliar effect, *p* < 0.0001; well x foliar, *p* < 0.001). There were no significant differences among the experimental treatments in seedling biomass at the main harvest (shoots 5.8 ± 0.3 mg, *p* = 0.7; roots 1.7 ± 0.1 mg, *p* = 0.4; Online Resource 2).

**Fig. 3:**
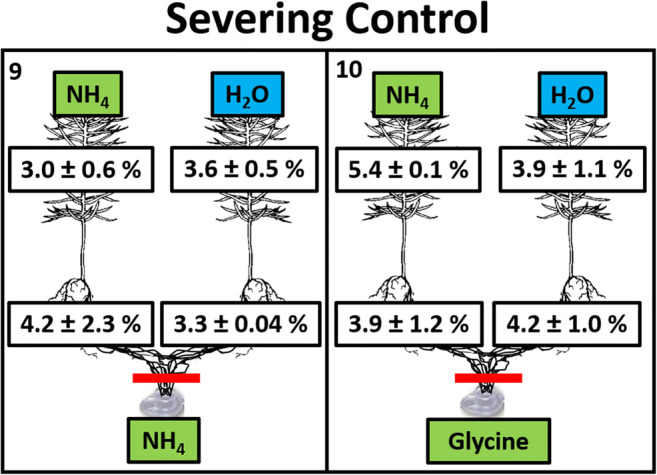
Nitrogen concentrations (%N; mean ± SD) of root and shoots of *P. contorta* seedlings after 70 days of growth followed by 3 days of foliage treatment (H_2_O or 4.7 mM NH_4_) and finally 2 days of hyphal well addition (9.46 mM N from glycine or NH_4_Cl). *n* = 4. Red lines indicate manual severing of hyphae at the time of N addition to the wells. Treatment numbers are in the top left corner of the boxes

The severing control microcosms had hyphal connections between the seedlings and wells cut immediately before the NH_4_ or glycine additions to the wells on day 73. The shoots and roots of these seedlings (Fig. 3) had similar N concentrations to the early harvest seedlings (Fig. 2a; treatments 1 + 2 vs. 9 + 10; shoots, *p* = 0.8; roots, *p* = 0.3; Online Resource 2). In other words, seedlings with severed hyphae showed no signs of N loss in response to N addition to the wells. Instead, they had approximately three times the shoot N concentrations as seedlings that were treated identically except for the severing of hyphae (compare treatments 3 and 4 in Fig. 2b with treatments 9 and 10 in Fig. 3).

### Nitrogen concentration in naturally occurring field seedlings

**Fig. 4:**
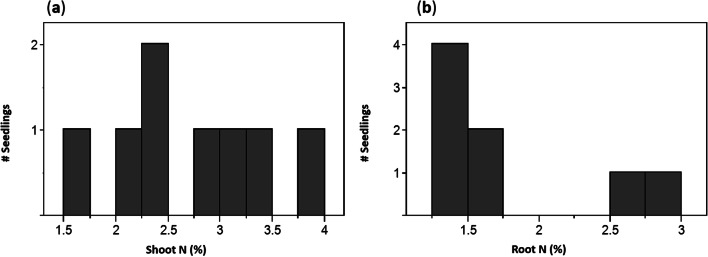
Frequency distribution of N concentrations in **a** shoots and **b** roots of eight 16- to 20-week-old naturally regenerating *P. contorta* seedlings from clearcut sites near Kelowna, British Columbia, Canada (elevation 1480 m)

The foliar N concentrations observed in the early harvest seedlings (treatments 1, 2; Fig. 2a) were considerably above those expected in foliage of mature lodgepole pine trees, according to nutritional guidelines for this species in British Columbia forests (Brockley 2001a). Because these experimental seedlings were considerably younger (10 weeks old) than trees used to generate the nutritional guidelines, we sampled naturally regenerating, 16- to 20-week-old *P. contorta* germinants from two recently logged sites in order to determine whether these tissue N concentrations were typical for seedlings of a relatively similar age growing in the field. All except one of the field seedlings had started to form ectomycorrhizas. Nitrogen concentrations in foliage of the naturally regenerating lodgepole pine seedlings covered a similar range to that seen in the experimental seedlings (Fig. 4). The highest foliar N observed in the field seedlings was nearly identical to that of the early harvest seedlings (approximately 4%), whereas the lowest was just slightly higher than those of seedlings that had experienced rapid N loss (approximately 1.2%). Nitrogen concentrations in roots of field seedlings tended to be slightly lower than shoots, with 75% of the roots between 1.4 and 1.7%. Two seedlings had higher N concentrations, but these were still slightly lower than the values observed in experimental seedlings.

### N budget

**Fig. 5:**
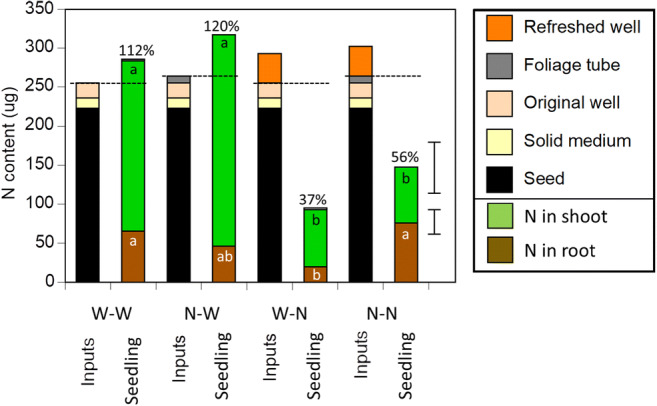
Sources of readily available N (Inputs) and the final distribution of N in shoots and roots of *P. contorta* seedlings at the 75-day harvest (Seedling). The first letter represents the foliage treatment (W = water; N = NH_4_); the second letter represents the well treatment (W = water; N = NH_4_ or glycine). For inputs, the foliage tube compartment was set at 9 μg because that was the amount, on average, taken up by the shoots. An additional 2.8 μg N was added to the W-W and W-N final distribution bars to account for N lost to the water applied to the foliage. Shoot and root N values are averages of the appropriate seedlings from the following treatments: W-W = treatments 5, 8 [*n* = 12]; N-W = treatment 5 [n = 4]; W-N = treatment 3, 4, 6, 7 [*n* = 32]; N-N = treatments 3, 4 [*n* = 16]. Horizontal lines above bars mark the sums of readily available N inputs for that treatment, assuming no N was absorbed from refreshed wells. See Online Resource 5 for N contents of less available sources such as agar and charcoal-infused filter paper. The percentages are the mean percentages of the sum of readily available N inputs found in each group of seedlings. The root mean square errors for the shoot and root dataset are shown as vertical bars. For each plant tissue, bars sharing a letter were not significantly different according to a Dunn nonparametric multiple comparison test

Nitrogen budgets were constructed to examine N contents of the seedlings and to compare these with the amount of readily available N to the seedlings (Fig. 5). Shoots from microcosms where the wells had been replenished with N at day 73 appeared to have lost substantial amounts of N relative to shoots from other microcosms (Fig. 5; Kruskal-Wallis *p* < 0.001, Online Resource 3), with no effect of N addition to the foliage. By contrast, the N content of roots was affected by both foliage and well treatments. While N addition to the wells appeared to trigger N loss from roots if the foliage had been exposed to only water (W-N vs W-W in Fig. 5), if the foliage had been exposed to N prior to the well treatment, root N content remained high (N-N in Fig. 5).

We considered readily available N sources in the microcosms to be seed N, N in the plate and well media, and N added to the foliage. The mean N content of seedlings with water added to the hyphal wells was slightly higher than the sum of those readily available sources (W-W and N-W in Fig. 5), suggesting that the extra N came from less readily available N, such as agar and the charcoal-infused filter papers (Online Resource 5). By contrast, only an average of 37 to 56% of the readily available N remained in seedlings in treatments where the wells were refreshed (W-N and N-N in Fig. 5). To be conservative, these estimates assume no uptake from the second application of N to the hyphal wells. Nevertheless, an average of 8 μg of the 19 μg ^15^N added to the wells was detected in shoot and roots, and if this 8 μg is added as an additional source of readily available N, the percentage of readily available N remaining in seedlings averaged 36% for treatments where the foliage was exposed to water and 54% for treatments where the foliage was exposed to ammonium. Seed N accounted for the majority of the N available to the seedlings, but in treatments where the foliage was exposed to water and hyphae were exposed to additional N, only 43% of seed N was retained by seedlings; for seedlings where the foliage was exposed to ammonium, only 66% of seed N remained after well media was refreshed with N. A very small amount of N was lost from foliage treated with water (2.8 ± 6.9 μg N); Online Resource 4), and this was added back in the N budget calculations but is barely visible in Fig. 5.

## Discussion

In many studies of EM seedlings, colonization by the EM fungus is associated with increased accumulation of N compared to non-mycorrhizal plants (e.g., Plassard et al. 1994; Peay et al. 2010; Jenkins et al. 2018). This is especially true when supplied N is in complex organic forms (Akroume et al. 2019; Nicolás et al. 2019), because most tree seedlings cannot access these without EM fungi (Abuzinadah et al. 1986). As a result, EM fungi are viewed as an essential conduit of N from soil to their plant partners (Smith and Read 2008). Nevertheless, especially under conditions of low-N supply, retention of N by EM fungi may result in N limitation for the phytobiont (Abuzinadah and Read 1989; Wallander 1995; Wallander et al. 1999; Hobbie and Colpaert 2003; Franklin et al. 2014). In N-limited forests, increasing transfer of plant carbon to EM fungi can result in decreased allocation of fungal N to the trees (Hasselquist et al. 2016; Högberg et al. 2017). The original goal of our study was to test whether the concentration of N in the foliage of a plant partner would also influence the rate of N transfer from its EM fungal symbiont. This would be predicted based on the positive relationship between foliar N concentration and carbon assimilation rates (Evans 1989). Although some N was absorbed from the solution applied to the foliage of our pine seedlings, it did not result in an increase in tissue N in either shoots or roots prior to the well treatments. Furthermore, ^15^N from the wells was detected in only two-thirds of the shoots, resulting in high variability in ^15^N values in the plant tissues. For these two reasons, we were not able to determine whether distribution of N by an EM fungus within a mycorrhizal network is influenced by the N status of its plant symbiont.

Even though we were not able to test our original hypothesis, we detected unique changes in N concentrations and contents of seedlings during the final stages of the experiment. To our knowledge, this is the first report of a rapid, major loss of N from EM seedlings: a 60% reduction in plant N content over 48 h, apparently signaled by a change in the environment of the fungus. However, it is not the first record of a reduction in seedling N over the first few months of growth. Zackrisson et al. (1997) measured the N status of natural *Pinus sylvestris* L. germinants over their first growing season in the field and found that seedlings experienced a 35% N loss compared to original seed reserves, except in the treatment where EM hyphae were severed. As in the current experiment, seedlings with severed hyphae in the Zackrisson et al. (1997) study retained all of their seed N. The absence of N loss by EM seedlings with severed hyphae in both studies indicates that the EM fungus was involved, but does not shed light on the mechanism by which the N was lost.

### Where did the nitrogen go?

Because there was very little or no N detected in the water-filled microcentrifuge tubes applied to the foliage (Online Resource 4), we can conclude that N was not lost from foliage. This means that the roots were the most likely exit point for N from the seedlings. Furthermore, roots of seedlings treated with foliar N retained more N than seedlings whose foliage was treated with water (Figs. 2b and 5). This could have been a result of movement of N from shoots to roots in seedlings where N was added to foliage, but there could be other explanations for the retention of N in those root systems. The retention of ^15^N in the shoots of most seedlings from plates with ^15^N-labeled hyphal wells suggests that some of this recently transferred N was incorporated into nonmobile pools and could not be re-translocated to the roots. Two mechanisms seem likely to be responsible for the altered N partitioning by the seedlings in response to hyphal exposure to higher N: increased rates of root exudation or transfer of N from seedling to EM fungus.

The composition of root exudates can change over several days in response to nutrient deficiency (Jones et al. 2004), with amino acids as a common component (Haichar et al. 2014). Under lab conditions, Abuzinadah and Read (1986) found non-mycorrhizal pine seedlings lost approximately 9% of the N originating from their seeds over a 70-day period. They attributed this loss to exudation from roots, triggered by N starvation. In our system, *P. contorta* seedlings had absorbed all of the readily available N in the microcosms (Fig. 5), so it is possible that they responded with increased root exudation even though N concentrations in plant tissues were still very high.

The other possibility is that N moved from the seedlings into the fungus. Although the seedlings were not N-limited, if the hyphae had taken up all of the N added to the well early in the experiment, then the fungus may have become N limited. In this case, the addition of N to the wells on day 73 could have stimulated increased metabolism and growth of the whole mycelium, similar to the “invigoration” of the mycelium observed by Perez-Moreno and Read (2000) when one portion of a *Paxillus involutus* mycelium reached a nutrient-rich patch of substrate. Ectomycorrhizal fungi can alter their metabolism within days of encountering a patch of nutrients (Tibbett and Sanders 2002), which would be consistent with the rapid response seen here. Another possibility is that the seedlings, and therefore the fungus, were carbon limited because of low rates of diffusion of CO_2_ into the Petri plates and relatively low irradiance in the growth cabinet. When starved for carbon, EM fungi can undergo autophagy by increasing production of N transporters in order to reallocate nutrients within the mycelium (Ellström et al. 2015). Increased metabolism or growth of the fungus in response to replenishment of the well could have triggered a renewed demand for carbon from the host. If that need for carbon was met through transfer of amino acids from the host, the N-replete germinants could have acted as N sources rather than N sinks.

Even though the function of N transporters at EM root-fungal interfaces is not completely understood (Nehls and Plassard 2018), the movement of N from plant to fungus could be explained under current models. For example, Garcia et al. (2016) speculate that NRT2 nitrate transporters could be a mechanism by which mycobiont and phytobiont compete for N in the root-fungal interface depending on plant and fungal nutrient status. This could explain the data of Hobbie et al. (2008) that were consistent with movement of N from plant to fungus when N was supplied as nitrate. Amino acids such as glutamine were traditionally considered likely candidates for N transfer from fungus to plant (Martin et al. 1986; Smith and Smith 1990; Nehls and Plassard 2018). In that scenario, a shuttle of amino acids back and forth across the root-fungal interface was hypothesized to provide carbon skeletons for transamination in fungal tissues (Martin et al. 1986; Smith and Smith 1990). Increased efflux of glutamate from root cells into the interface would allow N uptake by the fungus. More recently, however, evidence is accumulating that ammonium is the form of N most commonly transported from fungus to plant, with some ammonia transported by major intrinsic proteins (MIPs; Nehls and Plassard 2018). Major intrinsic proteins are protein channels that transport substrates based on their concentration gradients. If fungal cells in the Hartig net suddenly became stronger sinks for N as hyphae began to repair and grow, consuming N for chitin and protein synthesis, the MIPs could have facilitated movement of N down a concentration gradient from the root cells, which had very high concentrations of N, to the fungus. Similarly, Lucic et al. (2008) detected a low-affinity H^+^-NH_4_^+^ symporter in mycorrhizal *Laccaria bicolor*, which may be upregulated under conditions of high external N (Nehls and Plassard 2018), such as was experienced by the *S. tomentosus* after well replenishment in our experiment. These are examples of molecular mechanisms that could explain how plant-fungal transfer of N might occur under the conditions described in this study and deserve further investigation.

Both of the hypothesized mechanisms, increased root exudation or transport into the fungus, would have increased N concentrations of the filter paper or solid media in the microcosms. Unfortunately, because the hyphae grew among cellulose fibers on filter paper, they could not be extracted to compare hyphal N concentrations among treatments. Furthermore, because the agar and charcoal-infused filter papers were such major pools of (relatively unavailable) N compared to other pools (> 2 mg N per plate; Online Resource 5), we could not detect any differences in their N concentrations among treatments (data not shown). Consequently, we could not distinguish between the two hypotheses, and further research will be required in order to determine the mechanism by which such a large amount of N was lost by the seedlings.

### Ecological implications

The experiment reported here was performed under artificial conditions in the lab using a single species of EM fungus. Nevertheless, Zackrisson et al.’s (1997) observations indicate that a loss of N from first-year pine seedlings may be facilitated by fungal symbionts under field conditions as well. Although fungal-mediated N loss is counterintuitive for a symbiosis assumed to be based on mutual supply of limiting resources, the explanation may lie in the very high tissue N concentrations in young pine germinants and the early stage of mycorrhiza formation in this system. Both the experimental, and some of the field, seedlings in our study had N concentrations (4%) that were three times the minimum concentration considered to be adequate for foliage of mature pine trees in British Columbia (1.35%; Brockley 2001a). Under such conditions, partitioning some N to EM hyphae or the rhizosphere would not be expected to inhibit seedling growth. It would be a reasonable evolutionary strategy, especially if the result was an increased supply of N to the seedling over a relatively short time period (days to weeks). Loss of N, either through root exudates or transfer of N directly to an EM fungus, might act as a form of N priming by the seedling, similar to that documented for carbon. In carbon priming, soil microorganisms and fungi are unable to mineralize soil organic matter optimally unless an initial supply of labile carbon is provided via litterfall, root exudates, or plant symbioses (Hamer and Marschner 2005a). Similarly, priming with N would enable rhizosphere microorganisms to increase their production of excreted enzymes, giving them a significant resource exploitation advantage over other soil microbes (Hamer and Marschner 2005b; Chen et al. 2014). Root exudates are known to influence the composition of rhizosphere microbial communities in a manner than enhances future N uptake by the plant (Haichar et al. 2014). It is important to acknowledge that the fungus used here, *Suillus tomentosus*, produces copious amounts of extramatrical hyphae; other EM fungal species may not be such strong sinks for carbon or N.

An N priming mechanism would be especially beneficial for EM symbionts of lodgepole pine seedlings germinating under natural disturbance conditions in the field. Lodgepole pine seeds are adapted to germinate after wildfires (Anderson 2003). Severe wildfires kill trees in the preexisting stand and remove the N-rich forest floor. Under these conditions, most EM fungal species colonize pine germinants from spores (Glassman et al. 2016), meaning that the fungi would not have access to carbon and nutrients from a mycelial network associated with larger trees. Because spores have such small N reserves to support initial hyphal growth, a young seedling could be a source of N for EM fungi germinating after wildfire. Ectomycorrhizas were just forming in our system, a time when nutrient demand by the fungus would be high and changes in gene expression by the host, including some membrane transport proteins, is extensive (Heller et al. 2008). Importantly, even after losing N, the seedlings in our study had foliar N concentrations just slightly below the adequate range (Brockley 2001a) but still well within the range for survival. Therefore, by releasing some N into the soil or to EM symbionts, pine seedlings could increase the survival of soil microbes that would ultimately increase N supply, without compromising their own survival.

## Electronic supplementary material

ESM 1 (PDF 33.6kb)

ESM 2 (PDF 152 kb)

ESM 3 (PDF 74 kb)

ESM 4 (PDF 95 kb)

ESM 5 (PDF 135 kb)

## Data Availability

The datasets generated during and/or analyzed during the current study are available from the corresponding author on reasonable request.
