# Supplementary material for: Rapid nitrogen loss from ectomycorrhizal pine germinants signaled by their fungal symbiont
Source: Mycorrhiza. 2020 May 3;30(4):407–17. doi: 10.1007/s00572-020-00959-7 (PMC7314718; doi:10.1007/s00572-020-00959-7)
Supplement: Supplementary file 1 — (PDF 33.6kb) [file 572_2020_959_MOESM1_ESM.pdf]

**Online Resource 1**

Article title: Rapid nitrogen loss from ectomycorrhizal pine germinants signalled by their fungal symbiont

Journal: Mycorrhiza

Authors: Joshua M Smith, Matthew D Whiteside and Melanie D Jones

Corresponding author: Melanie D Jones

Biology Department and Okanagan Institute of Biodiversity Resilience and Ecosystem Services, University of British Columbia, Okanagan campus, Kelowna, British Columbia, V1V 1V7 Canada

melanie.jones@ubc.ca

**Online Resource 1.** $^{15}N$ concentrations in shoots of *Pinus contorta* associated with *Suillus tomentosus*.

| Treatment Number | Foliar Treatment | Hyphal Well[3] | Harvest Time (d) | Hyphae | $^{15}N$ Analyses Replicates (Foliar Application): Shoot ($H_2O$) | Shoot (N) | Root ($H_2O$) | Root (N) | $^{15}N$ at% (± SD): Shoot ($H_2O$) | Shoot (N) | Root ($H_2O$) | Root (N) |
|---|---|---|---|---|---|---|---|---|---|---|---|---|
| 1 | Differential[1] | N/A | 73 | Intact | 3 | 3 | 5[4] | - | 0.36498 ± 0.00044 | 0.36467 ± 0.00044 | 0.36529 ± 0.00082[4] | - |
| 2 | $H_2O$[2] | N/A | 73 | Intact | 6 | - | 4 | - | 0.36456 ± 0.00059 | - | 0.36546 ± 0.00065 | - |
| 3 | Differential[1] | $NH_4$ | 75 | Intact | 8 | 8 | 7 | 7 | 0.37013 ± 0.00714 | 0.37459 ± 0.02246 | 0.40144 ± 0.03624 | 0.44986 ± 0.13613 |
| 4 | Differential[1] | Glycine | 75 | Intact | 8 | 8 | 7 | 6 | 0.40763 ± 0.06895 | 0.41788 ± 0.12623 | 0.77019 ± 0.53239 | 0.96079 ± 1.14465 |
| 5 | Differential[1] | $H_2O$ | 75 | Intact | 4 | 4 | 3 | 3 | 0.36481 ± 0.00044 | 0.36478 ± 0.00092 | 0.36525 ± 0.00094 | 0.36518 ± 0.00082 |
| 6 | $H_2O$[2] | $NH_4$ | 75 | Intact | 8 | - | 7 | - | 0.37173 ± 0.00967 | - | 0.39652 ± 0.03413 | - |
| 7 | $H_2O$[2] | Glycine | 75 | Intact | 8 | - | 6 | - | 0.36550 ±0.00237 | - | 0.37652 ± 0.01550 | - |
| 8 | $H_2O$[2] | $H_2O$ | 75 | Intact | 8 | - | 5 | - | 0.36485 ± 0.00069 | - | 0.36476 ± 0.00079 | - |
| 9 | Differential[1] | $NH_4$ | 75 | Severed | 4[4] | - | 4[4] | - | 0.36890 ± 0.00473[4] | - | 0.40152 ± 0.03464[4] | - |
| 10 | Differential[1] | Glycine | 75 | Severed | 5[4] | - | 4[4] | - | 0.36532 ± 0.00083[4] | - | 0.36819 ± 0.00521[4] | - |

[1]Microcosms where the foliage of one of the two *P. contorta* seedlings was treated with 4.7 mM-N $NH_4Cl$ and the other received deionized $H_2O$.

[2]Microcosms where the foliage of both seedlings received $H_2O$.

[3]At 73 d; 9.46 mM-N $NH_4Cl$ ($98^{+}$ at% $^{15}N$), 9.46 mM-N glycine ($99^{+}$ at% $^{15}N$), or deionized $H_2O$ were added to wells accessible by hyphae but not roots; N/A indicates treatments that were harvested before N additions were made to the hyphal wells.

[4]Analyses where differential foliar applications were merged due to low replicate counts.
