# Supplementary material for: Rapid nitrogen loss from ectomycorrhizal pine germinants signaled by their fungal symbiont
Source: Mycorrhiza. 2020 May 3;30(4):407–17. doi: 10.1007/s00572-020-00959-7 (PMC7314718; doi:10.1007/s00572-020-00959-7)
Supplement: Supplementary file 2 — (PDF 152 kb) [file 572_2020_959_MOESM2_ESM.pdf]

Online Resource 2

Article title: Rapid nitrogen loss from ectomycorrhizal pine germinants signalled by their fungal symbiont

Journal: Mycorrhiza

Authors: Joshua M Smith, Matthew D Whiteside and Melanie D Jones

Corresponding author: Melanie D Jones

Biology Department and Okanagan Institute of Biodiversity Resilience and Ecosystem Services, University of British Columbia, Okanagan campus, Kelowna, British Columbia, V1V 1V7 Canada

melanie.jones@ubc.ca

Online Resource 2. Results of linear and linear mixed effects model analyses

Type of Analysis	Variable	Plant Part	Treatments	Source/Effect	Sum of Squares	d.f.	Mean Square	F_s	P
Linear model with one fixed factor and plate as a random factor	%N	Shoot	1	plate	0.22247	1	0.22247	0.5723	0.5043
				foliar treatment	0.40056	1	0.40056	1.0305	0.3848
				residuals	1.16615	3	0.38872		
		Root	1	plate	0.10723	1	0.10723	0.713	0.4873
				foliar treatment	0.63870	1	0.63870	4.2472	0.1755
				residuals	0.30077	2	0.15038		
Linear model with one fixed factor; values averaged across seedlings on the same plate	%N	Shoot	Trmt 1 seedlings treated with water vs Trmt 2 averaged per plate	neighbour	0.09879	1	0.09879	0.2392	0.6504
				residuals	1.65202	4	0.413		
			6,7,8	well treatment	23.89680	2	11.94840	145.89	<0.0001
				residuals	0.73710	9	0.08190		
			9,10	well treatment	1.91900	1	1.91896	2.1583	0.2017
				residuals	4.44550	5	0.88909		
			1+2, 9+10	severing treatment	0.03000	1	0.03	0.041	0.844

				residuals	8.09900	11	0.7363		
		Root	Trmt 1 seedlings treated with water vs Trmt 2 averaged per plate	neighbour	0.13568	1	0.13568	0.898	0.4132
				residuals	0.45325	3	0.15108		
			6,7,8	well treatment	11.72400	2	5.8621	79.351	<0.0001
				residuals	0.591	8	0.0739		
			9,10	well treatment	0.0039	1	0.0039	0.0027	0.9609
				residuals	5.6773	4	1.4193		
			1+2, 9+10	severing treatment	0.79120	1	0.7912	1.141	0.313
				residuals	6.24100	9	0.6935		
Linear mixed effects models with well and foliar treatments as fixed factors and plate as a random factor	%N	Shoot	3,4,5	foliar treatment	0.04400	1	0.04350	0.3438	0.5653
				well treatment	42.80700	2	21.40350	168.9781	<0.0001
				foliar X well	0.02719	2	0.01340	0.1059	0.9001
			9,10	foliar treatment	0.34850	1	0.34850	0.5583	0.4886
				well treatment	4.13860	1	4.13860	6.6308	0.0497
				foliar X well	2.44060	1	2.44060	3.9103	0.10492

		3+4,5,9+10	foliar treatment	0.05650	1	0.05650	0.2783	0.6037
			well/severing treatment	40.26500	2	20.13250	99.1601	<0.0001
			foliar X well	0.00620	2	0.00310	0.0150	0.9851
	Root	3,4,5	foliar treatment	14.74715	1	14.74715	33.2222	<0.0001
			well treatment	4.40910	2	2.20455	4.9664	0.0146
			foliar X well	9.34963	2	4.67482	10.5314	0.0004
		9,10	foliar treatment	0.12950	1	0.12950	0.0662	0.8097
			well treatment	0.17179	1	0.17179	0.0878	0.7818
			foliar X well	0.70024	1	0.70024	0.3578	0.5819
		3+4,5,9+10	foliar treatment	3.75980	1	3.75980	6.2475	0.0172
			well/severing treatment	14.79410	2	7.39705	12.2929	<0.0001
			foliar X well	13.55510	2	6.77755	11.2633	0.0002
Biomass	Shoot	3-10	foliar treatment	0.0986	1	0.0986	0.0146	0.9042
			well/severing treatment	36.309	3	12.1031	1.791	0.1576
			foliar X well	4.012	3	1.3373	0.1979	0.8975
	Root	3-10	foliar treatment	0.011412	1	0.011412	0.4225	0.5227
			well/severing treatment	0.016363	3	0.005454	0.2019	0.8943
			foliar X well	0.178294	3	0.059431	2.2004	0.1178
