# Supplementary material for: Rapid nitrogen loss from ectomycorrhizal pine germinants signaled by their fungal symbiont
Source: Mycorrhiza. 2020 May 3;30(4):407–17. doi: 10.1007/s00572-020-00959-7 (PMC7314718; doi:10.1007/s00572-020-00959-7)
Supplement: Supplementary file 3 — (PDF 74 kb) [file 572_2020_959_MOESM3_ESM.pdf]

Online Resource 3

Article title: Rapid nitrogen loss from ectomycorrhizal pine germinants signalled by their fungal symbiont

Journal: Mycorrhiza

Authors: Joshua M Smith, Matthew D Whiteside and Melanie D Jones

Corresponding author: Melanie D Jones

Biology Department and Okanagan Institute of Biodiversity Resilience and Ecosystem Services, University of British Columbia, Okanagan campus, Kelowna, British Columbia, V1V 1V7 Canada

melanie.jones@ubc.ca

Online Resource 3. Results of Kruskal-Wallis and Dunn tests

Variable	Plant part	Treatments	Output	Value		
			Kruskal-Wallis		Dunn Test	P adj.
at% 15N averaged per plate	Shoot	3,4,5,6,7,8	ChiSquare	10.159	gly - H2O	0.1290
		well treatment	d.f.	2	gly - nh4	0.1352
			P	0.006223	H2O - nh4	0.0048
	Root	3,4,5,6,7,8	ChiSquare	11.174	gly - H2O	0.0066
		well treatment	d.f.	2	gly - nh4	0.8579
			P	0.0037	H2O - nh4	0.0052
at% 15N	Shoot	3,4	ChiSquare	0.6876		
		foliage treatment	d.f.	1		
			P	0.407		
		3,4	ChiSquare	0.2785		
		well treatment	d.f.	1		
			P	0.5977		
	Root	3,4	ChiSquare	0.0024		
		foliage treatment	d.f.	1		
			P	0.9613		
		3,4	ChiSquare	1.1397		

		well treatment	d.f.	1		
			P	0.2857		
					Dunn Test	P. adj
Total N	Shoot	See Fig. 5	ChiSquare	25.5730	NN-NW	0.0207
			d.f.	3	NN-WN	1.0000
			P	<0.0001	NW-WN	0.0231
					NN-WW	0.0005
					NW-WW	0.9629
					WN-WW	0.0001
	Root	See Fig. 5	ChiSquare	24.0050	NN-NW	1.0000
			d.f.	3	NN-WN	0.0001
			P	<0.0001	NW-WN	0.3586
					NN-WW	0.9502
					NW-WW	0.8882
					WN-WW	0.0060
