# Supplementary material for: Rapid nitrogen loss from ectomycorrhizal pine germinants signaled by their fungal symbiont
Source: Mycorrhiza. 2020 May 3;30(4):407–17. doi: 10.1007/s00572-020-00959-7 (PMC7314718; doi:10.1007/s00572-020-00959-7)
Supplement: Supplementary file 4 — (PDF 95 kb) [file 572_2020_959_MOESM4_ESM.pdf]

Online Resource 4.

Article title: Rapid nitrogen loss from ectomycorrhizal pine germinants signalled by their fungal symbiont

Journal: Mycorrhiza

Authors: Joshua M Smith, Matthew D Whiteside and Melanie D Jones

Corresponding author: Melanie D Jones

Biology Department and Okanagan Institute of Biodiversity Resilience and Ecosystem Services, University of British Columbia, Okanagan campus, Kelowna, British Columbia, V1V 1V7 Canada

melanie.jones@ubc.ca

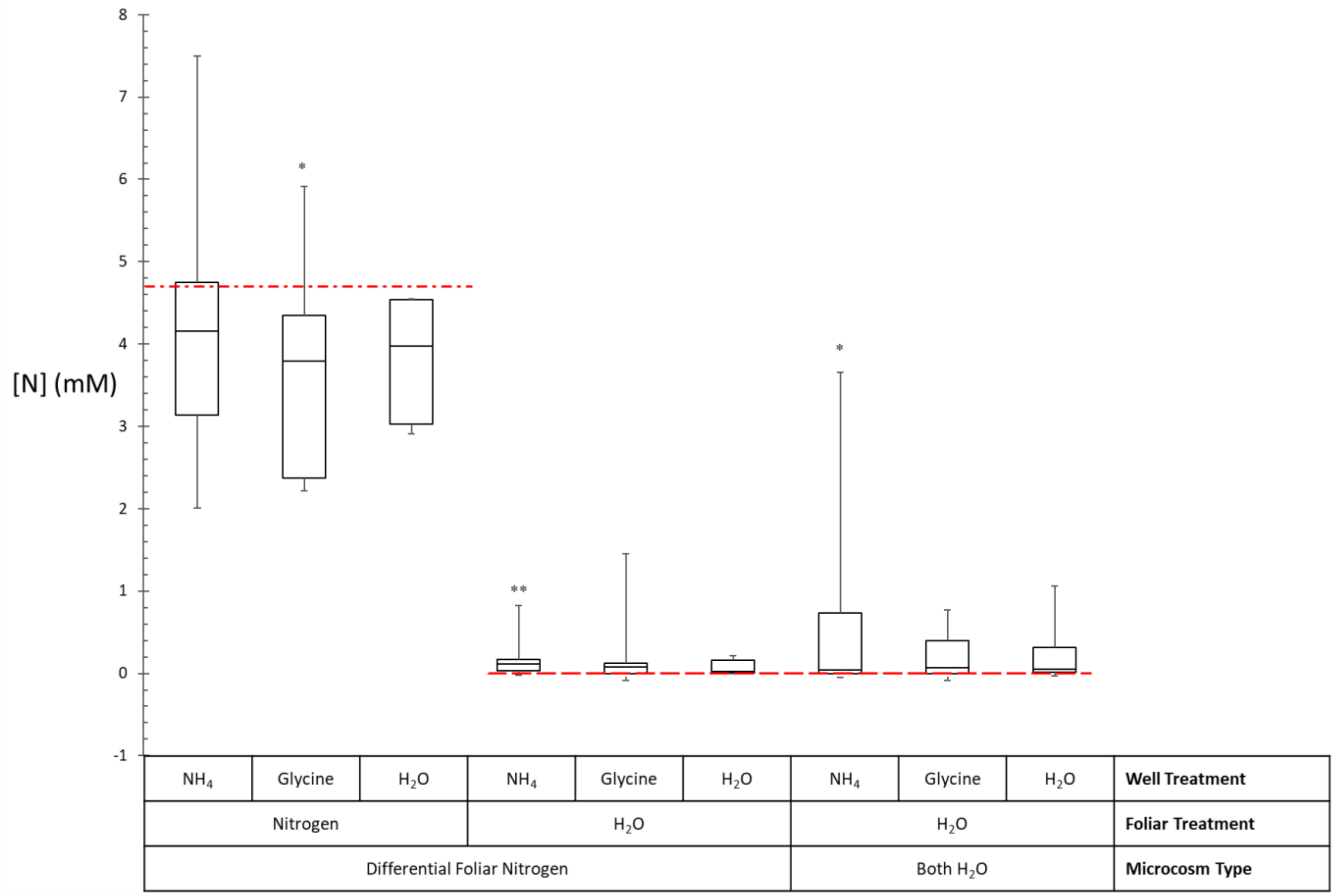

Online Resource 4. Final nitrogen concentration of the solutions applied to the seedling foliage, at harvest. The red line represents the original N concentration of the solutions (foliar N = 4.7 mM, foliar H_2O = 0 mM; 5d before harvest, 3d before well treatments applied). Significant differences from the original concentrations were tested by one-tailed T-test or non-parametric Wilcoxon Signed Rank T-test, where applicable (* = $p < 0.05$, ** = $p < 0.01$). Box plots show median (middle line), 1st and 3rd quartiles (box outlines) and minimum and maximum values (whiskers).
