# Supplementary material for: Rapid nitrogen loss from ectomycorrhizal pine germinants signaled by their fungal symbiont
Source: Mycorrhiza. 2020 May 3;30(4):407–17. doi: 10.1007/s00572-020-00959-7 (PMC7314718; doi:10.1007/s00572-020-00959-7)
Supplement: Supplementary file 5 — (PDF 135 kb) [file 572_2020_959_MOESM5_ESM.pdf]

Online Resource 5

Article title: Rapid nitrogen loss from ectomycorrhizal pine germinants signalled by their fungal symbiont

Journal: Mycorrhiza

Authors: Joshua M Smith, Matthew D Whiteside and Melanie D Jones

Corresponding author: Melanie D Jones

Biology Department and Okanagan Institute of Biodiversity Resilience and Ecosystem Services, University of British Columbia, Okanagan campus, Kelowna, British Columbia, V1V 1V7 Canada

melanie.jones@ubc.ca

Online Resource 5. Total N inputs into the microcosms.

N Source	N per Plate (µg ± SD)	N per Seedling (µg ± SD)
Seed	na	223 ± 48
Initial Well	38	19
Foliar N*	na	53
Replenished Well†	38	19
MMN plate media	26	13
Agar	1057	516
Charcoal-infused paper	1083 ± 80	542 ± 40

na = relevant to individual seedlings only

***** An average of 9 µg N was absorbed from the N applied to foliage

† An average of 8 µg N from ^{15}N was found in seedlings with access to the second application of N to the hyphal wells.
